# Chemical and mechanical influence of root canal irrigation on biofilm removal from lateral morphological features of simulated root canals, dentine discs and dentinal tubules

**DOI:** 10.1111/iej.13399

**Published:** 2020-11-19

**Authors:** T. C. Pereira, R. J. B. Dijkstra, X. Petridis, P.K. Sharma, W. J. van de Meer, L. W. M. van der Sluis, F. B. de Andrade

**Affiliations:** ^1^ Department of Dentistry, Endodontics and Dental Materials Bauru School of Dentistry University of São Paulo Bauru Brazil; ^2^ Center for Dentistry and Oral Hygiene University Medical Center Groningen University of Groningen Groningen The Netherlands; ^3^ Department of Biomedical Engineering University Medical Center Groningen University of Groningen Groningen The Netherlands; ^4^ Department of Orthodontics University Medical Center Groningen University of Groningen Groningen The Netherlands

**Keywords:** biofilm, confocal laser scanning microscopy, optical coherence tomography, polysaccharides, RISA, sodium hypochlorite, ultrasound

## Abstract

**Aim:**

To investigate the anti‐biofilm efficacy of irrigation using a simulated root canal model, the chemical effect of irrigants against biofilms grown on dentine discs and their impact on biofilm viscoelasticity, the efficacy of the irrigants in decontaminating infected dentinal tubules and the capacity of bacteria to regrow.

**Methodology:**

Biofilm removal, viscoelastic analysis of remaining biofilms and bacterial viability were evaluated using a simulated root canal model with lateral morphological features, dentine discs and a dentinal tubule model, respectively. Experiments were conducted using a two‐phase irrigation protocol. Phase 1: a modified salt solution (RISA) and sodium hypochlorite (NaOCl) were used at a low flow rate to evaluate the chemical action of the irrigants. Ultrasonic activation (US) of a chemically inert solution (buffer) was used to evaluate the mechanical efficacy of irrigation. Phase 2: a final irrigation with buffer at a high flow rate was performed for all groups. Optical coherence tomography (OCT), low load compression testing (LLCT) and confocal scanning laser microscopy analysis were used in the different models. One‐way analysis of variance (anova) was performed for the OCT and LLCT analysis, whilst Kruskal–Wallis and Wilcoxon ranked tests for the dentinal tubule model.

**Results:**

US and high flow rate removed significantly more biofilm from the artificial lateral canal. For biofilm removal from the artificial isthmus, no significant differences were found between the groups. Within‐group analysis revealed significant differences between the steps of the experiment, with the exception of NaOCl. For the dentine discs, no significant differences regarding biofilm removal and viscoelasticity were detected. In the dentinal tubule model, NaOCl exhibited the greatest anti‐biofilm efficacy.

**Conclusions:**

The mechanical effect of irrigation is important for biofilm removal. An extra high flow irrigation rate resulted in greater biofilm removal than US in the artificial isthmus. The mechanical effect of US seemed to be more effective when the surface contact biofilm–irrigant was small. After the irrigation procedures, the remaining biofilm could survive after a 5‐day period. RISA and NaOCl seemed to alter post‐treatment remaining biofilms.

## Introduction

Bacteria tend to grow in biofilms, adhering to a surface or liquid interface and co‐adhering to each other (Kolenbrander *et al*. 2010). In biofilms, microorganisms are protected by the extracellular polymeric substance (EPS) against chemical and mechanical stresses (Flemming *et al*. 2007) imposed by cleaning procedures and disinfectants (Stewart & Franklin 2008). The root canal wall is a surface where bacteria can adhere and biofilm can develop when the root canal system is infected (Chávez de Paz 2007, Ricucci *et al*. 2009). If the root canal is infected, biofilm will also be present in lateral morphological features such as lateral canals, fins, isthmuses and the dentinal tubules (Peters *et al*. 2001, Ricucci *et al*. 2013). To eradicate biofilms from these areas is a challenge. These lateral morphological features are not reached by instrumentation (Peters *et al*. 2001, Ricucci *et al*. 2013). In this respect, getting more insight into the fate of the remaining biofilm after mechanical or chemical challenges during root canal irrigation is warranted (remaining biofilm).

The aims of root canal irrigation are the chemical dissolution or disruption and the mechanical detachment of pulp tissue, dentine debris and smear layer (instrumentation products), microorganisms (planktonic or biofilm) and their products from the root canal wall and their removal from the root canal system. The chemically active sodium hypochlorite (NaOCl) is the most popular irrigation solution due to its bactericidal and anti‐biofilm effect and its capacity to inactivate endotoxins (Sarbinoff *et al*. 1983, Spratt *et al*. 2001, Silva *et al*. 2004, Tawakoli *et al*. 2017). Moreover, it has an excellent tissue dissolving ability (Abbott *et al*. 1991), being able to dissolve necrotic tissue (Naenni *et al*. 2004) and the organic compounds of smear layer (Tartari *et al*. 2016). However, NaOCl cannot dissolve inorganic tissue (Sen et al. 1995) and seems to have difficulty fully penetrating the biofilm (van der Waal *et al*. 2014, 2016, Petridis *et al*. 2019a), especially biofilms with a high bacterial density and less EPS (Busanello *et al*. 2019, Petridis *et al*. 2019a). Laboratory investigations have shown that NaOCl has intratubular disinfection ability in root canal models in bovine and human teeth (Arias‐Moliz *et al*. 2014, Baron *et al*. 2016, Morago *et al*. 2016, Giardino *et al*. 2018, Rodrigues *et al*. 2018, Liu *et al*. 2019, Morago *et al*. 2019). However, it is not known whether NaOCl irrigation is effective in removing biofilms with high bacterial density from lateral morphological features in a root canal model.

Modified salt solution, also called ‘RISA’, is a hypertonic salt solution with a high pH which can kill bacteria, inactivate and detach biofilm (van der Waal *et al*. 2011, 2014, de Almeida *et al*. 2016). RISA was developed to be used either as irrigant solution or intracanal medication during treatment exerting antimicrobial action based on a multiple hurdle effect, namely using an osmotic effect and weak acid as hurdles (van der Waal *et al*. 2015, De Almeida *et al*. 2016). Biofilm inactivation and removal from complex‐shaped areas as the root canal system have been demonstrated in an *ex vivo* study (van der Waal *et al*. 2015). Also, in an *in situ* model, RISA was able to generate a porosity in the bacteria cell membranes and a shrinkage of the biofilm, due to its hypertonic nature. When compared to NaOCl in a narrow channel model, RISA was associated with greater biofilm diffusion (van der Waal *et al*. 2017). However, there is no information in the literature on the ability of RISA to remove biofilm from lateral morphological features in a root canal model or from dentinal tubules when used as a root canal irrigant. Moreover, its influence on the viscoelastic properties of remaining biofilm has not been described, as opposed to the viscoelastic profile of biofilms submitted to NaOCl treatment investigated in previous studies (Busanello *et al*. 2019, Petridis *et al*. 2019a,b).

To improve the mechanical and chemical effect of the irrigation procedure, ultrasound has been used (Verhaagen *et al*. 2014, Robinson *et al*. 2018). This results in a more effective mechanical removal of bacteria, dentine debris and organic tissues (van der Sluis *et al*. 2007). Furthermore, several studies have shown an increase of the chemical effect of NaOCl in combination with ultrasound (Sabins *et al*. 2003, van der Sluis *et al*. 2005, 2007, Robinson *et al*. 2018). However, no study has evaluated the typical mechanical efficacy of ultrasound in biofilm removal from lateral morphological features in a root canal model nor on the effect of bacterial activity in human dentinal tubules.

Biofilm is considered as a recalcitrant structure to remove (Peterson *et al*. 2015). In addition, root canal anatomy can be extremely complicated. Therefore, it is generally accepted that complete removal of biofilm from the root canal system is not possible. As a consequence, it is important to know how treatment protocols influence the biofilm remaining after treatment. In addition, comparing the composition of the remaining biofilm directly after the disinfection procedure with the composition of the remaining biofilm after a 5‐day period without any extra source of nutrition could give more insight on the fate of the remaining biofilm persisting chemical disinfection.

To address the above‐mentioned topics biofilm removal, bacterial viability and changes in the polysaccharidic content of the biofilm matrix after irrigation with RISA, 2% NaOCl, buffer solution and ultrasonically activated buffer solution were evaluated. For this purpose, three different experimental models were employed. The aim of the first model was to evaluate biofilm removal from lateral morphological features in a simulated root canal model (artificial lateral canal or isthmus‐like structures) by means of optical coherence tomography (OCT). The aim of the second model was to evaluate biofilm removal by means of OCT and the viscoelastic properties of the remaining biofilms by means of low load compression testing (LLCT) after 30 s of static irrigant application on biofilms developed on dentine discs (Busanello *et al*. 2019). The aim of the third model was to evaluate bacterial viability and changes in the polysaccharidic content of the biofilm matrix of biofilms developed in a dentinal tubule model both directly after irrigation procedures (Giardino *et al*. 2018) as well as after 5 days in situ without nutrition.

The null hypothesis was that there is no difference in the four irrigation protocols regarding biofilm removal, inactivation and regrowth of bacteria in the biofilm, biofilm thickness reduction and viscoelastic properties of the remaining biofilm.

## Materials and methods

### Artificial root canal and dentine disc models

#### Bacterial strains and growth conditions

The clinical isolates *Streptococcus oralis* J22 and *Actinomyces naeslundii* T14V‐J1 were grown as described previously (Busanello *et al*. 2019, Petridis *et al*. 2019). Briefly, the bacteria were streaked on blood agar plates and a single colony was used to inoculate 10 mL modified brain–heart infusion broth (BHI; 37.0 g L^−1^ BHI, 1.0 g L^−1^ yeast extract, 0.02 g L^−1^ NaOH, 0.001 g L^−1^ vitamin K1, 5 mg L^−1^ L‐cysteine‐HCl, pH 7.3; BHI; Oxoid Ltd., Basingstoke, UK). Subsequently, *S. oralis* were cultured at 37 °C for 24 h in ambient air and *A. naeslundii* at 37 °C for 24 h in an anaerobic chamber (pre‐cultures). Pre‐cultures were used to inoculate 250‐mL modified BHI (1 : 20 dilution) and grown for 16 h (main cultures). Bacteria were harvested by centrifugal force (6350 ***g***) and washed twice in sterile adhesion buffer (0.147 g L^−1^ CaCl_2_, 0.174 g L^−1^ K_2_HPO_4_, 0.136 g L^−1^ KH_2_PO_4_, 3.728 g L^−1^ KCl, pH 6.8). The bacterial pellets were suspended in 10 mL sterile adhesion buffer and sonicated intermittently in ice water for 3 × 10 s at 30 W (Vibra cell model 375; Sonics and Materials Inc., Newtown, CT, USA) to break bacterial chains. Subsequently, bacteria were counted using a Bürker‐Türk counting chamber (Marienfeld‐Superior, Lauda‐Königshofen, Germany) and both suspensions were diluted in adhesion buffer in order to prepare a dual‐species bacterial suspension containing a concentration of 6 × 10^8^ bacteria mL^−1^ for *S. oralis* and 2 × 10^8^ bacteria mL^−1^ for *A. naeslundii*.

#### Preparation of the root canal model and dentine discs

**Figure 1:**
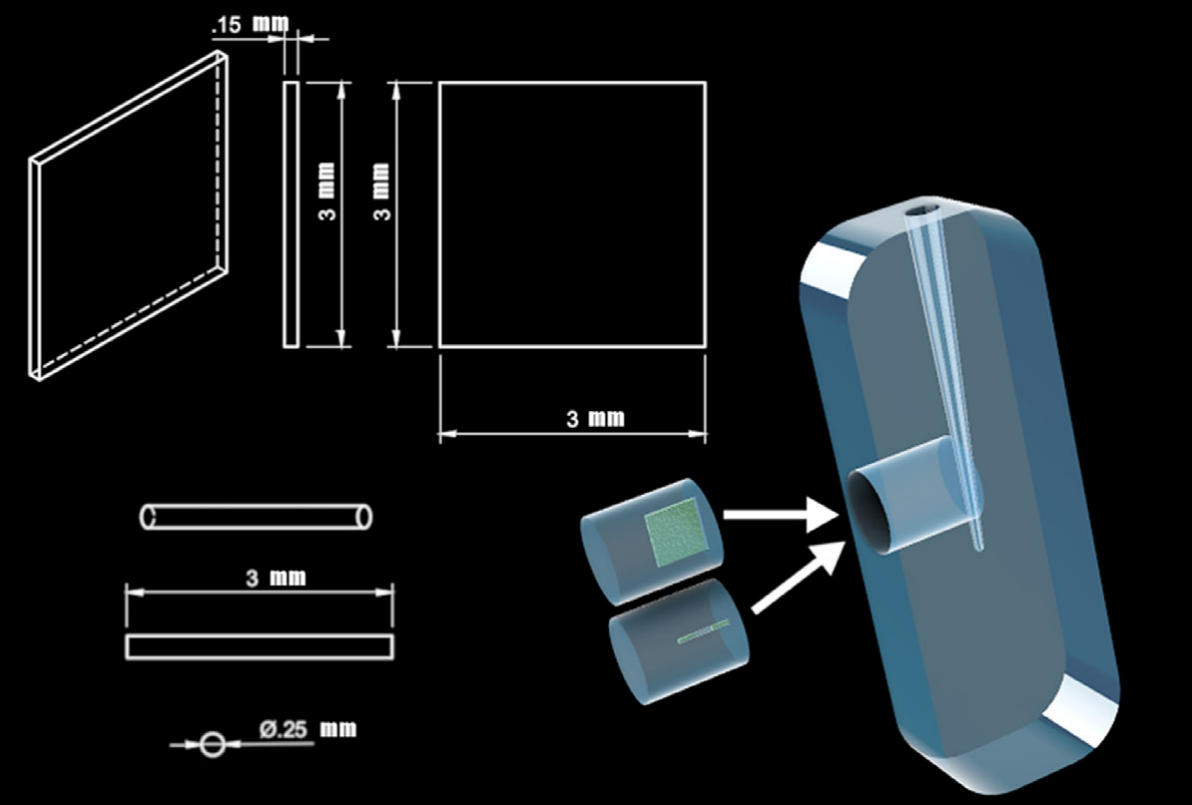
Schematic drawing of the artificial root canal and the plugs with isthmus‐like and lateral canal‐like structures.

**Figure 2:**
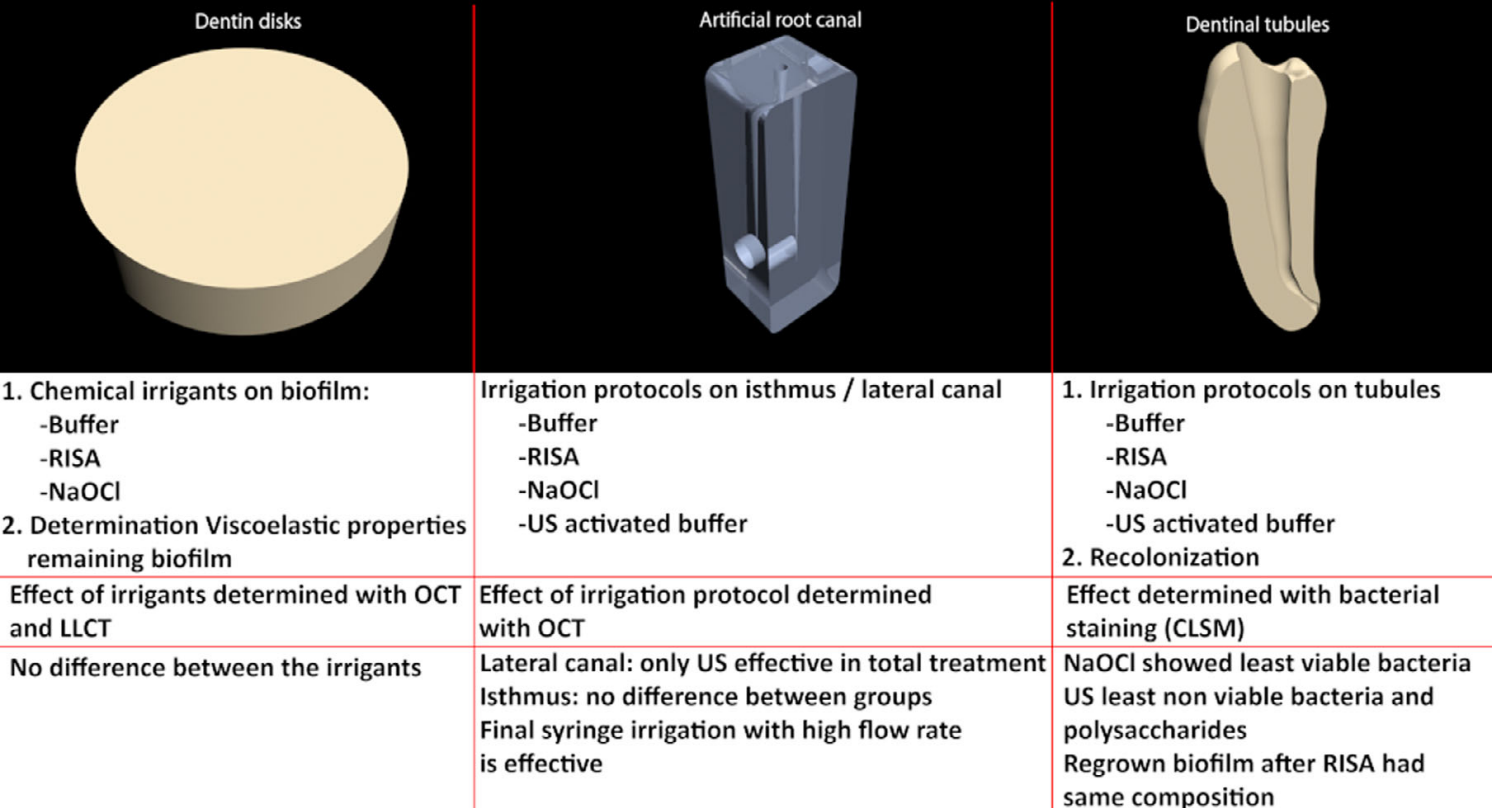
Representation of PDMS root canal model, dentine discs and dentine tubules model and their respective irrigation protocols and analysis.

Transparent PolyDiMethylSiloxane (PDMS; PolyDiMethylSiloxane; Sylgard 184, Dow‐Corning, Midland, MI, USA) root canal models, with a small plug (*R* = 2.5 mm) in the apical area perpendicular to the root canal, was created using a D‐size finger spreader (Dentsply Sirona, Ballaigues, Switzerland) as described Macedo *et al*. (2014; Fig. 1). PDMS plug inserts with anatomical features resembling an isthmus or lateral canal were created using moulds consisting of a thin metal strip (width 3 mm, thickness 0.15 mm, length 3 mm, total volume 1.35 mm^3^) or a small cylinder (length 3.0 mm and thickness 0.25 mm, total volume 0.29 mm^3^), respectively, as described before (Figs 1 and 2).

Dentine discs were prepared from the crown of freshly extracted human molars. Dentine cylinders were obtained using a diamond coated core drill (6 mm – CARAT N.V. Westerlo). Next, the cylinders were cut in 1.5‐mm‐thickness discs with the aid of a water‐cooled diamond blade (IsoMet, Diamond Wafering blades 102 × 0.3 mm; Buehler, Lake Bluff, IL, USA) mounted in a circular cutting machine. The dentine discs were treated 5 min with 17% EDTA, in a sonication bath to ensure removal of the smear layer (Fig. 2).

#### Biofilm growth in PDMS inserts and on dentine discs

Biofilms with a structure mimicking oral biofilms were developed (He *et al*. 2013). For that purpose, steady‐state biofilms were grown using a constant depth film fermenter (CDFF) in which a constant dropwise supply of nutrients combined with a repeated cycle of compression/scraping leads to a dental plaque‐like bacterial dense biofilm (Kinniment *et al*. 1996, Rozenbaum *et al*. 2019). The PDMS inserts and the dentine discs were coated with whole human saliva. The saliva coating consisted of freeze‐dried whole human saliva pooled from at least 20 volunteers of both genders (saliva was collected in agreement with the guidelines set out by the Medical Ethical Committee of the University Medical Centre Groningen, Groningen, the Netherlands, approval letter 06‐02‐2009) dissolved in 20 mL adhesion buffer (1.5 g L^−1^), stirred for 2 h and centrifuged at 10 000 ***g***, 10 °C, for 5 min. Both the inserts and the dentine discs were exposed to the reconstituted saliva for 14 h, at 4 °C, under static conditions. Subsequently, the inserts and dentine discs were transferred to the CDFF table at a depth of 250 μm, to ensure growth of biofilm with a standardized thickness. Dropwise application of 100 mL dual‐species bacterial suspension over 1 h at a constant slow rotation of the CDFF table ensured inoculation of the inserts and dentine discs. Rotation was stopped and bacteria were allowed to adhere for 30 min to the saliva‐coated dentine discs and inserts. Subsequently, rotation was resumed and a continuous supply of modified BHI (45 mL h^−1^) was started so biofilms could develop over the next 96 h at 37 °C.

### Dentinal tubule model

#### Allocation and preparation of the specimen (human mandibular incisors)

Eighty recently extracted human mandibular incisors were scanned, and the root canal anatomy was defined using a desktop microfocus computed tomographic scanner (SkyScan 1174v2; SkyScan, Kontich, Belgium). Thereafter, the teeth were equally distributed between four groups (*n* = 20) based on their root canal anatomy (volume). Teeth with small volumes and thin root canals, suggesting calcification or tubular sclerosis, were discarded. Subsequently, the teeth were immersed for 12 h in 1% NaOCl for surface disinfection. An access cavity was made, and the root canals were irrigated and instrumented with Prodesign Logic rotary files (Easy Equipamentos Odontológicos, Belo Horizonte, MG, Brazil). Working length was determined by measuring the length of a size 15 K‐file (Dentsply Sirona), when just extruding through the apical foramen (visual observation by eye) and then subtracting 1 mm from the measurement obtained. Subsequently, the teeth were submitted to three ultrasonic baths of 10 min each with 1% NaOCl, 17% EDTA (to remove smear layer) and saline solution to neutralize the NaOCl and EDTA (Marinho *et al*. 2015). Following, the external surface of the roots was covered with two subsequent coatings of red nail polish (Colorama, Rio de Janeiro, RJ, Brazil). The teeth were autoclaved (Cristófoli, Campo Mourão, PR, Brazil) at 121 °C for 24 min, inserted in sterile BHI culture media (brain–heart infusion, Difco, Detroit, MI, USA) and submitted to an ultrasonic bath for 10 min for maximum penetration of the culture broth into the dentinal tubules. All experiments were conducted under aseptic conditions in a laminar flow chamber to prevent airborne bacterial contamination.

#### Dentinal tubule contamination

The bacterial reference strain *Enterococcus faecalis* (ATCC 29212) was used for the experiments. The colonial morphology and Gram stain were verified to confirm strain’s purity by means of microbiological culture and microscopy, respectively. Stain’s purity analysis was repeated throughout the experiment. The microorganisms were cultivated in BHI, making a pre‐ and a main culture before establishing an inoculum for sample contamination. Dilutions were made based on the absorbance value, obtained by SF325NM spectrophotometer (Bel Photonics do Brazil Ltda, Osasco, Brazil) at 540 nm, to a concentration of 3 × 10^8^ CFU mL^−1^. The root canals and dentinal tubules were contaminated over a 5‐day period, in BHI medium at 37 °C, according to the protocol of Andrade *et al*. (2015) and the sequence of centrifugation steps of Ma *et al*. (2011).

### Irrigation protocols

#### Root canal model

After 96 h of biofilm formation, the inserts containing biofilm‐filled lateral canals or isthmuses were removed from the CDFF, carefully placed in the root canal model and imaged using the OCT (Thorlabs Ganymede II; Newton, NJ, USA). The purpose of this image acquisition was to determine the biofilm volume present inside the lateral canal or isthmus prior to any irrigant application. Next, 40 samples were randomly allocated to the following four groups (*n* = 10): Group 1: control (buffer solution: 0.147 g L^−1^ CaCl_2_, 43.5 g L^−1^ K_2_HPO_4_, 34.0 g L^−1^ KH_2_PO_4_, 3.728 g L^−1^ KCl, pH 6.8; B); Group 2: RISA (R); Group 3: 2% NaOCl; Group 4: ultrasonic activated irrigation (US) with a nonchemical active buffer solution. Each sample from the four groups was submitted to a two‐stage irrigation protocol.

In stage one, irrigation was performed using a 5‐mL irrigation syringe (Ultradent Products Inc., South Jordan, UT, USA) with a 30‐G irrigation needle (Endo‐Eze; Ultradent Products Inc.) at a flow rate of 0.05 mL s^−1^. During irrigation, the needle was placed 2‐mm coronal from the apical end‐point of the root canal and the irrigant solutions were continuously released during 30 s by in and outward movements of 5 mm amplitude. The 2% NaOCl concentration was obtained from a standard solution of NaOCl 12‐15% (Sigma‐Aldrich, St Louis, MO, USA) by means of iodometric titration before every experiment was conducted. For group 4, the buffer solution in the root canals was 3 × 20 s ultrasonically activated, following a clinical protocol previously described (Duque *et al*. 2017). Prior to each activation, root canals were gently replenished with 0.5 mL of buffer solution. The US was performed with a 25 mm long, size 25, IrriSafe file (Satelec Acteon, Merignac, France), energized with a commercial endodontic ultrasound device (Varios 350 Optic Complete System ‐ Osaka, Japan). The instrument was centred as much as possible and up and down movements were made in coronal direction from 2 mm coronal from the apical end‐point. At the end of irrigation, OCT images were again acquired in order to determine the post‐treatment biofilm volume remaining.

Stage two consisted of a final rinse with buffer at a high flow rate of 0.167 mL s^−1^ for 30 s (5 mL) to test its mechanical effect, and the specimens were again imaged with OCT.

#### Dentine disc model

In order to analyse only the chemical effect of the irrigant solutions on the biofilm, 30 dentine discs carrying CDFF‐grown were used. After 96 h of biofilm formation, biofilm‐carrying dentine discs were removed from the CDFF, carefully submersed in buffer solution and imaged with the OCT. The purpose of this image acquisition was to determine the biofilm thickness prior to any irrigant application. Next, the 30 discs were randomly allocated to the following three groups (*n* = 10): Group 1: control (buffer solution; B); Group 2: RISA (R); Group 3: 2% NaOCl.

With the aid of a pipette, 40 µL of each solution was gently dropped over the biofilm (no flow, static irrigant application; Busanello *et al*. 2019, Petridis *et al*. 2019a,b). The dentine discs were placed in an empty Petri dish (Sigma‐Aldrich, Merck KGgA, Darmstadt, Germany; 90 mm diameter × 15 mm height). After 30 s exposure to each irrigant, the biofilm‐carrying discs were again imaged with OCT in order to determine the post‐treatment biofilm thickness.

#### Dentinal tubule model

During the experimental procedures, the roots were placed in a sterilized metal device inside the laminar flow chamber. The samples were divided into four groups (*n* = 20) according to the irrigant used: Group 1: control (buffer solution; B), Group 2: RISA (R), Group 3: 2% NaOCl (NaOCl), Group 4: ultrasonic activated irrigation of a buffer solution (US). In all groups, the root canals were continuously irrigated with 1.5 mL of each irrigant for 30 s (0.05 mL s^−1^ flow rate) with a disposable plastic syringe with attached to it a 25‐mm stainless steel Endo‐eze 30‐gauge needle (Ultradent) positioned 2 mm short of working length (WL). In the US group, the buffer solution was three times ultrasonically activated for 20 s following a clinical protocol previously described (Duque *et al*. 2017). Prior to each activation, root canals were gently replenished with 0.5 mL of buffer solution. The US was performed with a 25 mm long, size 25 IrriSafe file (Satelec Acteon), energized with a commercial endodontic ultrasound device (Varios 350 Optic Complete System). The instrument was centred as much as possible and up and down movements were made in coronal direction from 2 mm coronal from the apical end‐point.

After having performed the experiments, the root canals were irrigated with 1.5 mL of buffer solution for 30 s (0.05 mL s^−1^ flow rate). Ten teeth from each group were used for confocal laser scanning microscopy (CLSM)‐based microbiological examination and the remaining teeth were used in the bacterial recolonization protocol below.

### Bacterial development 5 days after treatment (dentinal tubule model)

In 10 teeth from each group, selected based on an equal distribution of the root canal volume between the groups, the coronal opening and the apex were sealed with a restorative material (Villevie^®^, Dentalville, Joinville, SC, Brazil) after the irrigation procedure. In order to avoid an external contamination, the open jars containing the samples with the restorative material were exposed for 15 min to UV‐C germicidal lamp inside the laminar flow cabinet. In addition, the colonial morphology and Gram stain were also verified in this step of the experiment to confirm the strain’s purity by microbiological culture and microscopy, respectively. The teeth were placed in microtubes containing 1 mL of sterile BHI and incubated at 37 °C for 5 days to allow resumption of bacterial growth. After this period, the teeth were sectioned, stained and analysed by means of CLSM.

### Optical coherence tomography and biofilm removal assessment in root canal model

Quantitative image analysis for the biofilm removal from the artificial isthmus and lateral canal structures was performed by comparing pre‐, post‐ and final treatment images using ImageJ FIJI software program (National Institutes of Health, Bethesda, MD, USA). Three‐dimensional (3D) scans (750 × 373 pixels, field of view of 5.0 mm, refractive index 1.33) containing 750 slices each were acquired. The images were submitted to Otsu’s greyscale thresholding (Otsu 1979) which allowed for biofilm selection and background noise filtering. As a result, the volume of residing biofilm inside the isthmus and lateral canal was determined. Percentage removal was calculated by determining the difference in biofilm volume between pre‐treatment and post‐treatment or final irrigation.

### Optical coherence tomography and biofilm removal assessment in dentine disc model

Image analysis for dentine discs was performed using two‐dimensional (2D) high‐resolution OCT scans (5000 × 373 pixels, field of view 5 mm, refractive index 1.33) in order to determine biofilm thickness. With the aid of ImageJ FIJI software program and Otsu’s greyscale thresholding (Otsu 1979), the biofilm was separated from the background noise. Subsequently, the bottom and upper contour lines of the biofilm were defined by identifying the pixels in the image with a grey value exceeding the grey value threshold and connecting to neighbouring pixels. The mean biofilm thickness was calculated per vertical line scan (5000 px) based on the number of pixels between the bottom of the biofilm and the upper contour line. After this, the thickness reduction (biofilm height) was calculated after treatment for each specimen.

### Low load compression testing and assessment of biofilm viscoelastic properties in dentine disc model

Biofilms were submitted to a 20% deformation within 1 s, after which the deformation was held constant for 100 s (He *et al*. 2013). The relaxation was monitored over time and normalized over the cross‐sectional area of the plunger to calculate the induced stress. The percentage change in induced stress occurring within 100 s from its initial value was termed the percentage stress relaxation (R). The stress relaxation curves for each biofilm were modelled using a generalized Maxwell model, in which *E*(*t*) represents the total stress exerted by the biofilm divided by the imposed strain and it is expressed as the sum of four Maxwell elements, with a spring constant *E_i_*, and characteristic decay time, τ*_i_* (Busanello *et al*. 2019). The relative importance of each element was expressed as the percentage of its spring constant to the sum of the spring constants of all elements at *t* = 0. The elements derived were named corresponding with the outflow of water from the biofilm. Fast moving water or ‘free water’ and relatively slow‐moving water or ‘bound water’ being the E_1_ and E_2_ elements, EPSs being the E_3_ and bacterial rearrangement being the E_4_ (Busanello *et al*. 2019). Samples were submerged in adhesion buffer during measurements, and due to the sensitivity of the weight and to the duration of the measurements (100 s), a correction for water evaporation was applied.

### Confocal laser scanning microscopy image acquisition and analysis for dentinal tubule model

After irrigation, the roots were longitudinally sectioned using a circulating diamond blade saw (Erios, São Paulo, Brazil) under cooling with freshwater in such a way that every section contained a buccal and a lingual part. Then, one half of each root was placed in a 24‐well culture plate filled with 17% EDTA for 5 min and washed with 500 µL sterile saline to remove the smear layer resulting from the cutting. Next, 25 µL LIVE/DEAD^®^ BacLight™ bacterial viability dye (Invitrogen Molecular Probes, Eugene, OR, USA) was applied and the specimens were incubated in a dark environment for 20 min. With this protocol, bacteria with intact cell membrane are stained green and bacteria with compromised cell membrane red. Subsequently, excessive dye was removed and the specimens were stained with 25 µL of Calcofluor white M2R dye (Merck, Darmstadt, Germany) for 10 min to stain for polysaccharides in the biofilm matrix. The excessive dye was removed and the specimens were observed through the Leica TCS‐SPE CLSM (Leica Microsystems GmbH, Mannheim, Germany). Eight images were obtained for each root, four from the cervical and four from the apical thirds. Of these four images, two were made from the buccal side of the specimen and 2 from the lingual side. Of these two images, one represented a scan of the superficial tubules and the other the deeper aspect of the tubules. Following this protocol, the dentinal tubules were clearly visible using a 40× objective, scanning with a resolution of 1024 × 1024 pixels and a spacing of 1 µm. The images (275 µm × 275 µm) were acquired with the Leica Application Suite‐Advanced Fluorescence (LAS AF, Leica) program (Fig. 2).

After CLSM image acquisition, ImageJ software (FIJI ImageJ v 1 .50g, National Institutes of Health) was used to separate the bacterial contamination from the background fluorescence of the dentine (image analysis method developed specifically for the intratubular bacterial contamination). Using this method, the image stacks were analysed and the amount of ‘green’ (bacteria with intact cell membrane), ‘red’ (bacteria with compromised cell membrane) and ‘blue’ (biofilm matrix polysaccharides) present inside the dentinal tubules was evaluated (mean percentage of each component).

### Statistical analysis

The statistical analysis was performed using SPSS software (version 23.0 ‐ IBM Corp., Armonk, NY, USA). For the results obtained from the OCT analysis of the root canal and dentine disc models, paired sample *t*‐test and one‐way analysis of variance (anova) test with Tukey *post hoc* test were performed. For the results obtained from the LLCT analysis of the remaining biofilms on the dentine disc model, one‐way anova with Tukey HSD *post hoc* tests was performed. For the results obtained from the CLSM analysis of the dentinal tubule model, Kruskal–Wallis tests were used for the comparisons of the different treatments, as were Wilcoxon ranked test was used to compare the positions within each group.

## Results

### Root canal model

#### Biofilm removal from artificial lateral canal structures

**Figure 3:**
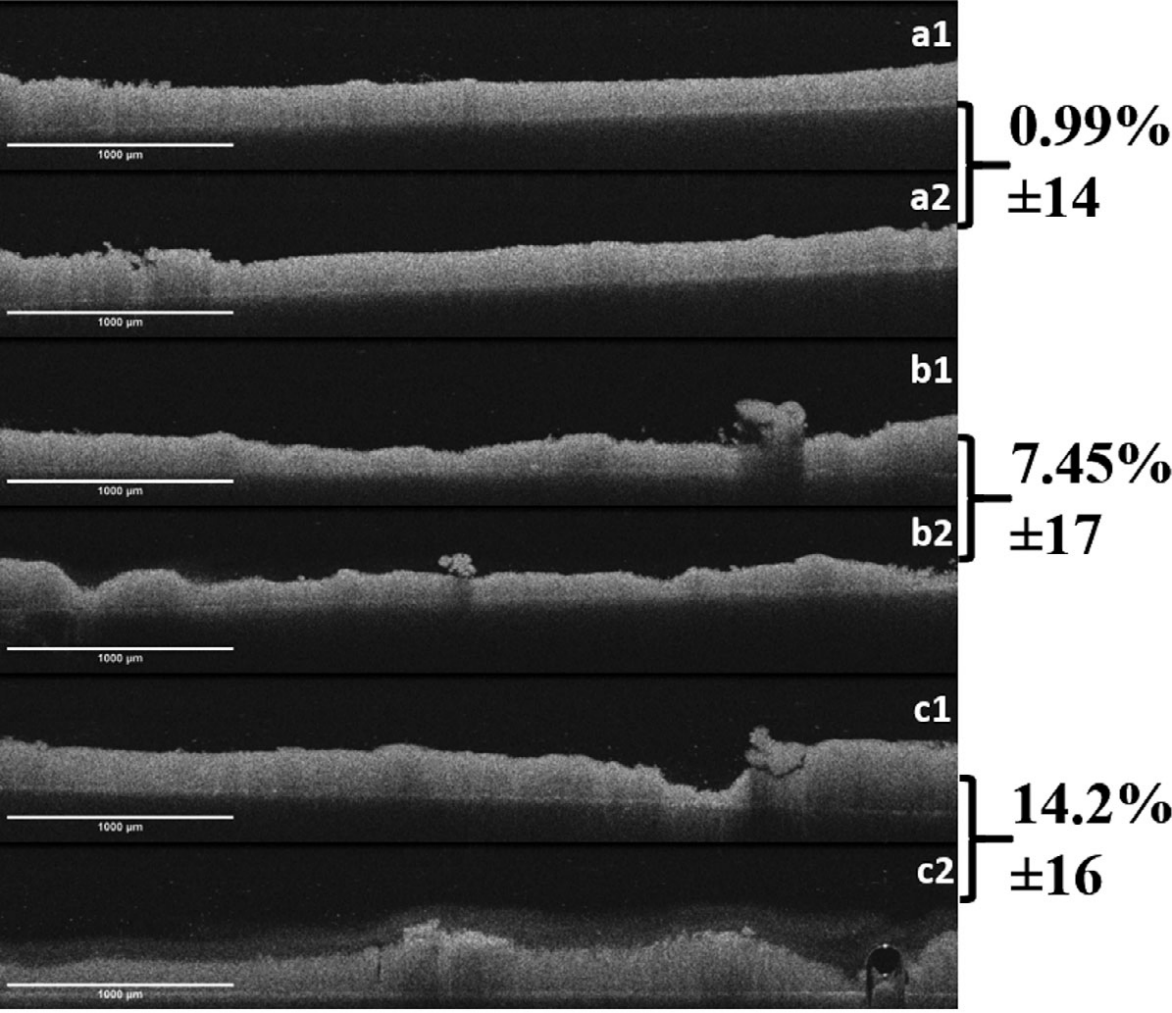
Mean (± SD) percentage of biofilm removal between pre‐ and post‐treatment (Stage 1), post‐treatment and final irrigation (Stage 2) and pre‐treatment and final irrigation (Total effect) for the lateral canal and isthmus‐like structures.

**Figure 4:**
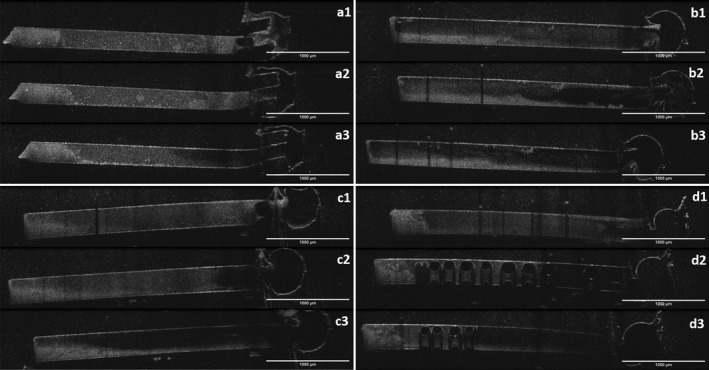
Pre‐treatment (1), post‐treatment (2) and after final irrigation (3) OCT images of biofilm removal in lateral canal model. (a) Control; (b) RISA; (c) 2% NaOCl; (d) US. Frontal 2D view of the lateral canal‐like structure, the round part in the right represents the main root canal.

The data are presented in Fig. 3. Between the different groups, no significant difference was detected. Within the groups, only US had significant difference in biofilm removal between the pre‐treatment and final irrigation (26.18% removal, *P* < 0.001) and post‐treatment and final irrigation (15.71% removal, *P* = 0.008; Fig. 4).

#### Biofilm removal from artificial isthmus structures

**Figure 5:**
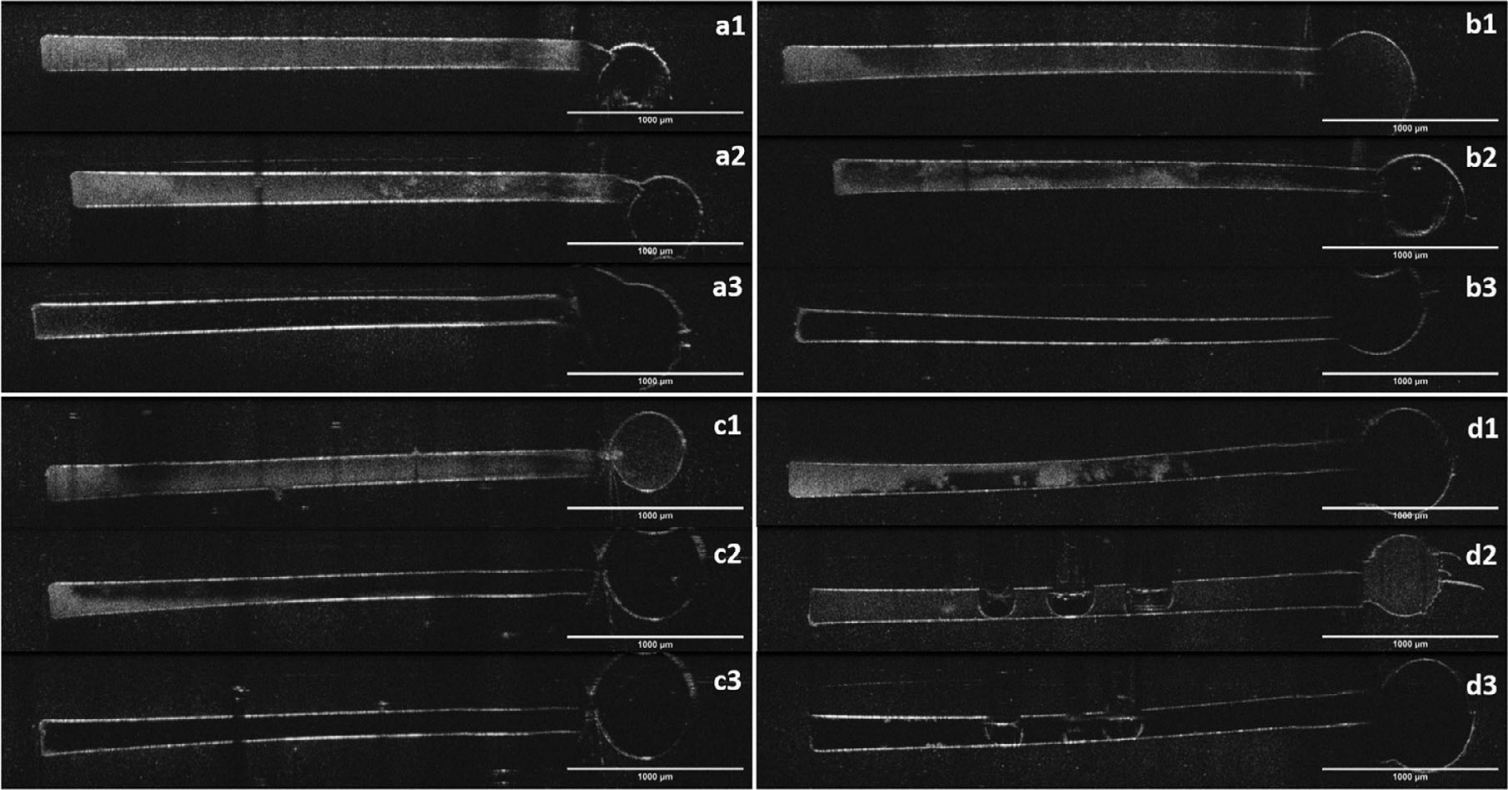
Pre‐treatment (1), post‐treatment (2) and after final irrigation (3) OCT images of biofilm removal in isthmus model. (a) Control; (b) RISA; (c) 2% NaOCl; (d) US. Frontal 2D view of the isthmus‐like structure, the round part in the right represents the main root canal.

The data are presented in Fig. 3. Between the groups, no significant differences were detected. Within the groups, all groups presented significant differences between the different steps of the experiment (pre‐treatment, post‐treatment and final irrigation), exception for NaOCl post‐treatment and final irrigation (Fig. 5).

### Dentine disc model

#### Biofilm removal

**Figure 6:**
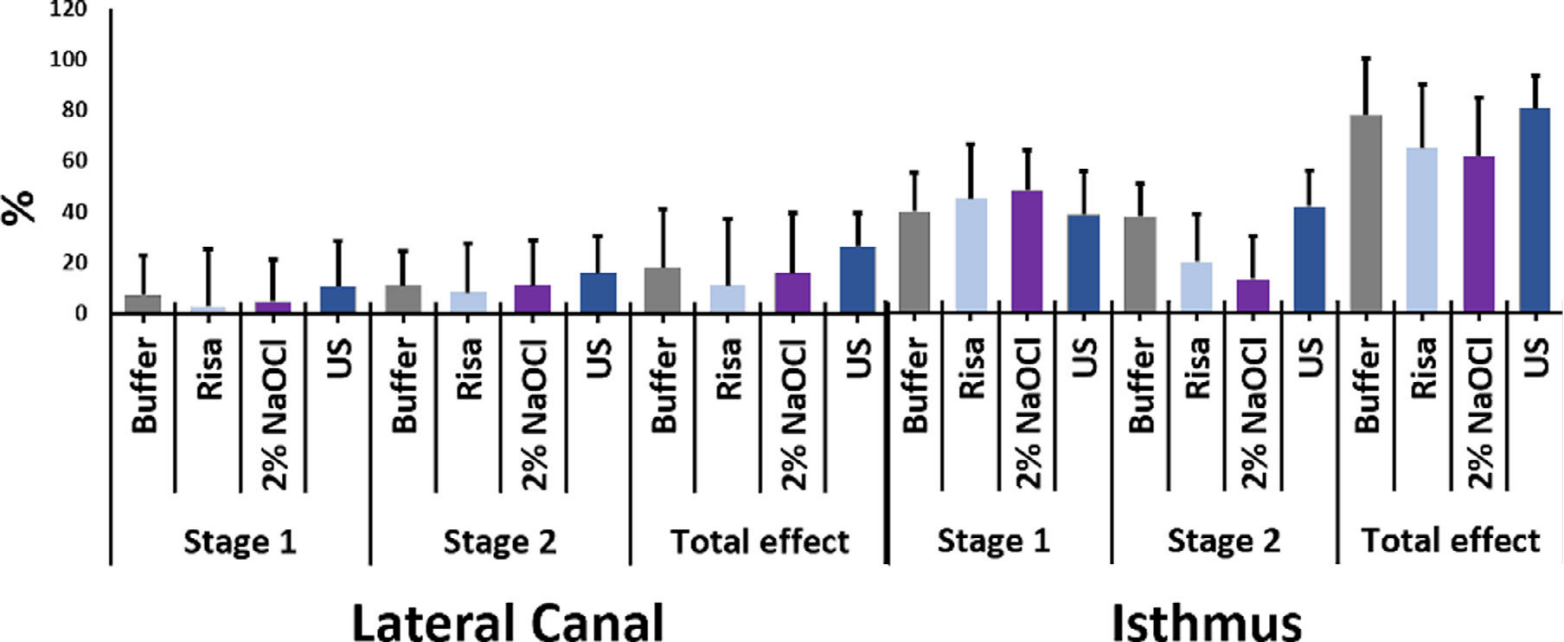
OCT images of biofilm on the dentine discs pre (1)‐ and post (2)‐direct contact with the irrigating solutions. (a) Control; (b) RISA; (c) 2% NaOCl. Biofilms on dentine discs (not visible, in black) are the less grey irregular structure.

The data are presented in Fig. 6. There was no significant difference between the groups. Within the groups, a significant reduction in biofilm thickness before and after 30 s of exposure only to NaOCl was noted (14.2% removal, *P* = 0.011). Figure 6 shows OCT images of biofilms pre‐ and post‐treatment with the irrigants employed.

#### Viscoelasticity of remaining biofilms

All data are presented in Table 1. No significant differences were detected between the irrigants employed.

**Table 1:** Stiffness, relaxation and relative importance of the four Maxwell elements after irrigation with different solutions

Groups	Stiffness (Pa)	Relaxation (%)	E1 (%)	E2 (%)	E3 (%)	E4 (%)
Buffer solution	1131 ± 1084	70.1 ± 15	42.2 ± 17	22.6 ± 6	4.3 ± 4	30.7 ± 17
R	1108 ± 1541	66.7 ± 18	28.4 ± 17	30.7 ± 12	6.7 ± 7	34.1 ± 19
2% NaOCl	436 ± 253	63.4 ± 17	35.3 ± 14	18.1 ± 13	10.1 ± 7	36.3 ± 17

Data refer to average ± SD (*n* = 5).

### Dentine tubule model

#### Initial treatment

The data are presented in Table 2. The NaOCl group had a significantly lower percentage of viable bacteria (16.1%) compared to the other groups, namely control (31.7%, *P* < 0.001), RISA (37.7%, *P* < 0.001) and US (53.1%, *P* < 0.001). US resulted in a significantly higher percentage of viable bacteria than the control (*P* < 0.001) and RISA (*P* = 0.027). For US, the percentage of nonviable bacteria was significantly lower than the NaOCl group (*P* < 0.001) and the control (*P* < 0.001). US had a significantly lower percentage of polysaccharide content (11.7%) than NaOCl (27%, *P* < 0.001) and RISA (20.2%, *P* = 0.001). The control group (15.3%) had a significant lower percentage of polysaccharide content compared to NaOCl (*P* = 0.001).

**Table 2:** Mean, SD and *P* values of the percentage of biovolume microorganisms with intact cell wall (LIVE), compromised cell wall (DEAD) and polysaccharides (EPS) after initial treatment (IT) and 5 days after treatment (5DAT) for all groups (*P* < 0.05)

	Buffer		R		NaOCl		US	
	IT	5DAT	IT	5DAT	IT	5DAT	IT	5DAT
LIVE	31.7 ± 25.2^A^	20.1 ± 17.9^ac^	37.7 ± 21.3^A^	35.9 ± 21.4^b^	16.1 ± 17.3^B^	23.9 ± 24.1^c^	53.1 ± 28.2^C^	28.6 ± 19.2^bc^
DEAD	48.4 ± 28.1^AB^	45.2 ± 23.9^a^	42.0 ± 19.8^AB^	42.0 ± 25.4^a^	47.4 ± 26.4^A^	42.8 ± 28.0^a^	32.9 ± 25.0^B^	57.1 ± 23.8^b^
EPS	15.3 ± 15.2^AB^	29.3 ± 14.3^a^	20.2 ± 11.2^BC^	19.5 ± 13.6^b^	27.0 ± 21.0^C^	20.7 ± 17.2^b^	11.6 ± 14.9^A^	11.7 ± 12.4^c^

Different capital and lowercase letters in the numbers indicate significant intergroup differences after initial treatment and after recolonization, respectively (*P* < 0.05).

#### CLSM analysis 5 days after treatment

The data are presented in Table 2. The control group presented 20.1% of viable bacteria, which was significantly less than US (28.6%, *P* = 0.044) and RISA (35.9%, *P* < 0.001). This last group also had a significantly higher percentage of viable bacteria compared to NaOCl (23.9%, *P* < 0.001). US displayed a significantly higher percentage of nonviable bacteria (57.2%) compared to the control (45.2%, *P* = 0.012), RISA (42%, *P* = 0.001) and NaOCl (42.8%, *P* = 0.003). US (11.7%) had the lowest percentage of polysaccharide content (11.7%) compared to the control (29.4%, *P* < 0.001), NaOCl (20.7%, *P* = 0.003) and RISA (19.5%, *P* = 0.010). Moreover, the control had the highest percentage of polysaccharide content that was signicantly different compared to NaOCl (*P* = 0.001) and RISA (*P* = 0.001).

#### Initial treatment versus 5 days after treatment

**Figure 7:**
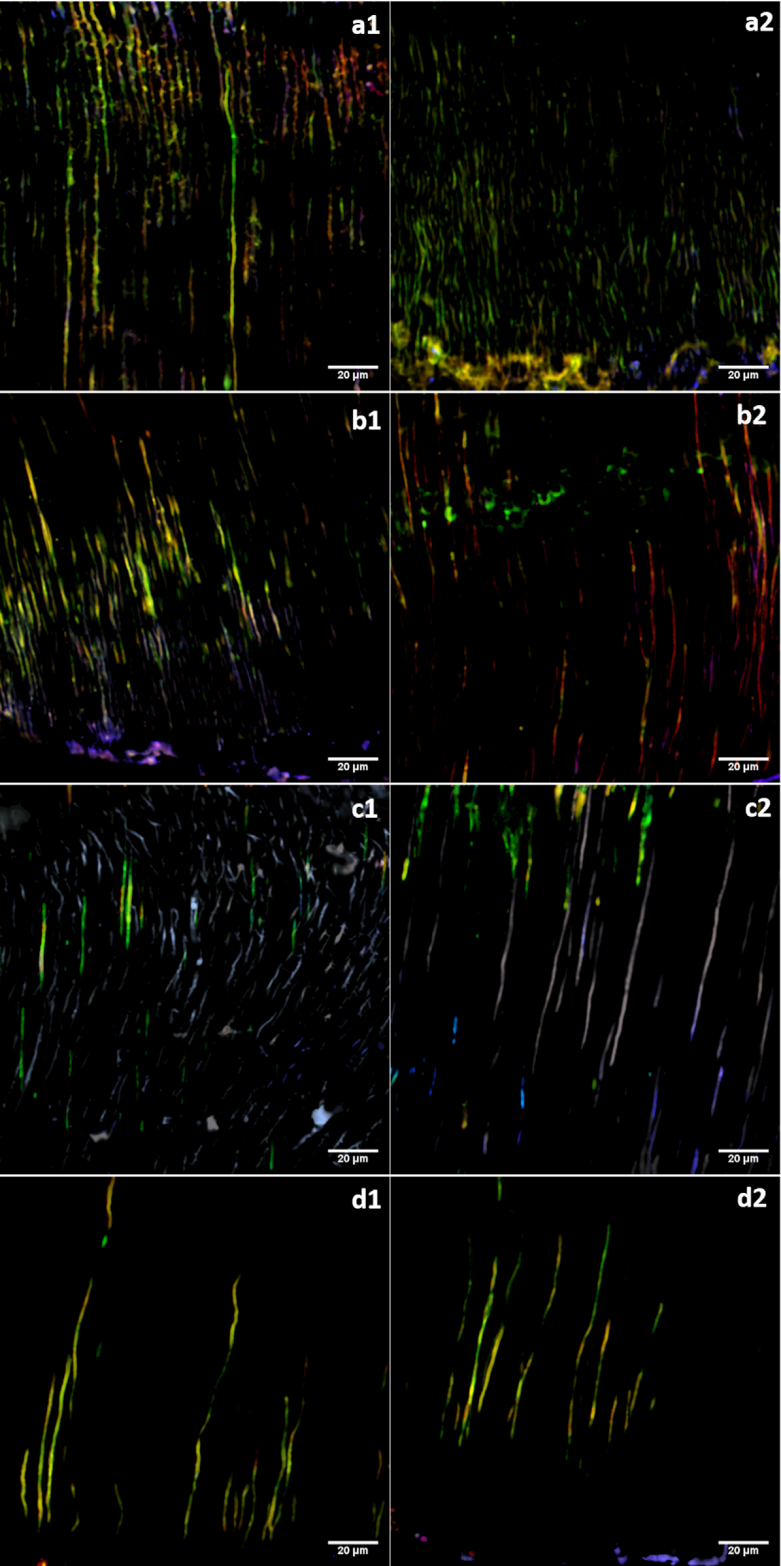
CLSM images of the analysis after the initial treatment (1)* and 5 days after treatment (2)*. (a) Control; (b) RISA; (c) 2% NaOCl; (d) US. *Images 1 and 2 do not correspond to the same dentine neither the same points of the dentine mass.

In the control and US, the percentage of viable bacteria noted in the tubules was reduced after recolonization from 31.7% to 20.2% (*P* = 0.000) and 53.1% to 28.6% (*P* = 0.002), respectively. The percentage of nonviable bacteria was significantly different only in the US group (32.9% vs. 57.2%, *P* < 0.001). A significant increase in the percentage of polysaccharide content was found only in the control group, from 15.3% (initial treatment; 15.3%) to 29.4% after recolonization (*P* < 0.001; Fig. 7).

## Discussion

### Root canal model with lateral morphological features

In the root canal model with lateral morphological features, the outcome measure was biofilm removal evaluated by OCT. OCT is a noninvasive imaging method enabling biofilm height determination, imaging of the biofilm structure and bacterial density as well as multiple assessments on the same biofilm sample (Wagner & Horn 2017, Busanello *et al*. 2019, Hou et al. 2019). In the present study, this longitudinal evaluation method provided information on biofilm removal from the artificial lateral canal and isthmus structures and allowed for each sample to be used as its own control. Thereby, individual variations in the biofilm structure and volume could be accounted for (Busanello *et al*. 2019). This is important in biofilm research as biofilm growth is difficult to standardize despite the standardized laboratory procedures employed (Swimberghe *et al*. 2019). However, OCT does not provide any information on bacterial vitality. Thus, inclusion of the dentinal tubule model in this study is considered an additional experimental method with high relevance. Furthermore, dentinal tubules can be seen as lateral morphological features with a significantly smaller diameter than the lateral canals in the root canal model.

For the root canal model, a dual‐species *in vitro* biofilm (*S. oralis* J22 and *A. naeslundii* T14V‐J1) was used instead of a hydrogel employed in previous studies (Macedo *et al*. 2014, Robinson *et al*. 2018), a methodological parameter that more closely approaches the clinical situation. Biofilms are not as homogeneous as hydrogels in terms of structure and also exhibit preferential growth in some locations and corners in the artificial lateral canals and the isthmus structures. With regard to the PDMS substrate used for the development of the lateral morphological features in the root canal model, it has been shown that biofilm adheres firmly to PDMS (Song & Ren 2014). This justifies its use as a substrate for biofilm growth, possibly making biofilm removal even more challenging compared to the clinical situation.

The bacterial species used for biofilm growth are often found in infected root canal systems (Chávez de Paz *et al*. 2003), whilst the viscoelastic properties of these biofilms resemble close those of an *in vivo* oral biofilm (He *et al*. 2013). Although the closed architecture and limited space of the artificial isthmus and lateral canal structures do not allow for direct evaluation of the viscoelastic properties of the biofilms developed inside, biofilms in the present study were grown based on previously established protocols resulting predictably in the formation of a rich‐cell biofilm with a dense basal layer (Busanello *et al*. 2019, Petridis *et al*. 2019a,b). In addition, the artificial isthmus in root canal model is a closed system (Fig. 1). This hampers biofilm removal induced by a steady‐state fluid flow, as opposed to artificial isthmi models with unblocked lateral openings at both sides. In the latter models, biofilms are more prone to irrigant flow‐supported removal. The model approximates clinical situations where isthmi may have one opening partially or completely occluded (e.g. debris accumulation).

The afore‐mentioned methodological parameters could account for the less biofilm removal observed in this study compared to the removal of hydrogel from the hydrogel‐filled lateral morphological features of root canal models utilized in other investigations (Macedo *et al*. 2014, Robinson *et al*. 2018). Also, they could explain why ultrasonic activation of a chemically inert buffer solution did not lead to increased biofilm removal from the artificial isthmus structure compared to the syringe buffer irrigation, even though the total time applied for irrigation in the ultrasonic group was longer. Contrary to the findings of the present study, previous studies have shown that ultrasound was effective in cleaning artificial isthmus structures (Macedo *et al*. 2014, Robinson *et al*. 2018). Differences in bacterial species composition and structure may have also accounted for this discrepancy (Busanello *et al*. 2019). Finally, in the present study only the mechanical aspect of ultrasound was examined, using a buffer as irrigant. It is known that ultrasound improves the chemical effect of NaOCl (synergistic effect; Moorer & Wesselink 1982).

Interestingly, for the artificial isthmus structures, the chemical effect of NaOCl and RISA was comparable to the mechanical effect of a low flow during syringe irrigation with buffer. Moreover, the mechanical effect of the buffer applied with a high flow rate in stage 2 removed even more biofilm than chemically active irrigants in stage 1. This indicates the importance of a high flow rate in removing biofilm from lateral morphological features. The penetration of irrigant depends on the anatomy of the root canal (Gulabivala *et al*. 2005). In open isthmi models, a steady jet of irrigant solution can be formed, promoting a slow and steady biofilm removal that does not occur in the lateral canals, resulting in more biofilm removal (Jiang *et al*. 2010, Verhaagen *et al*. 2014). The relatively short irrigant application time (30 s) compared to other *in vitro* studies may have influenced the results of the present study. Time is an important factor and could improve the chemical action of the irrigants (Petridis *et al*. 2019b).

In artificial lateral canal structures, hydrogel detaches more in fragments (Macedo *et al*. 2014, Robinson *et al*. 2018). Assuming the same pattern for biofilm detachment in the present, the mechanical effect of the lateral streaming produced by ultrasound had a significantly stronger impact than the chemical effect of NaOCl and RISA and the mechanical effect of syringe irrigation with buffer at low flow rate. Ultrasonic activation of the irrigant produces an oscillatory component near the file and a steady component further away (Verhaagen *et al*. 2014), whilst irrigant flow can be guided as the flow is strongest in the direction of the amplitude of the file (Jiang *et al*. 2010). Interestingly, the mechanical effect of a final flow with a high flow rate using syringe irrigation in stage 2 resulted in significant biofilm removal compared to stage 1. Arguably, the mechanical effect of the lateral streaming produced by ultrasound removed biofilm from the lateral canal close to the main canal and the high flow rate of the final flow resulted in removal from the deeper layers of the biofilm, which were probably already loosened by the ultrasound. It has been shown that after 20 s approximately a plateau effect is reached for the effectivity of ultrasound in hydrogel removal from artificial lateral canal structures (Macedo *et al*. 2014). This plateau effect could also account for the observations in the present study.

### Dentine disc model

RISA and NaOCl did not remove significantly more biofilm compared to the control. Within the groups, significantly increased biofilm removal compared to the biofilm prior to irrigation was observed only for NaOCl group. This is in line with the findings from the root canal model. The viscoelastic properties of the remaining biofilms after exposure to RISA or NaOCl were not significantly different from the control. This indicates that irrigant diffusion into the biofilm was not such that could alter the structure of the biofilm. The short exposure time (30 s) may account for this finding. A longer contact time may thus be needed for further irrigant diffusion in the bacterial dense biofilms used in the present study.

### Dentinal tubule model

In the dentinal tubule model, bacterial viability and the polysaccharidic content in the remaining biofilms and remaining biofilms 5 days after treatment were examined by CLSM. With CLSM, it is possible to image optical sections of the dentine mass at various depths up to 200 µm that are stacked and produce a three‐dimensional reconstructed image. Using fluorescent dyes, the architecture and spatial distribution of the biofilm inside the dentinal tubules are visualized. A semiquantitative analysis is feasible and both biofilm removal and bacterial viability inside the dentinal tubules can be assessed (Swimberghe *et al*. 2019). In this model, *Enterococcus faecalis* was used because of its ability to deeply penetrate the dentinal tubules and adhere to collagen (Love 2001). In addition, these bacteria have the capacity to regrow as they are able to achieve a viable but not culturable state (VBNC) by activating a starvation response under stress conditions (Heim *et al*. 2002), being suitable to regrow. The dentinal tubule model employed in the present study has been previously demonstrated to be an appropriate method to evaluate the effectiveness of the antimicrobial agents used in the endodontic treatment against bacteria inside the tubules (Andrade *et al*. 2015, Arias *et al*. 2016, Rodrigues *et al*. 2018). After irrigation with NaOCl, significantly less viable bacteria were seen compared to RISA and buffer, with the latter not being significantly different from each other. The effect of NaOCl comes in agreement with findings from earlier studies (Arias‐Moliz *et al*. 2014, Rodrigues *et al*. 2018). In contrast to earlier studies however, RISA was not more effective than the control (van der Waal *et al*. 2015, 2017, de Almeida *et al*. 2016). In this study, exposure time was shorter than compared to the previously mentioned investigations (30 s instead of 2 min or 1 h). This suggests that longer exposure times may be necessary for RISA to show enhanced anti‐biofilm efficacy. Also, different biofilms were used.

Ultrasonic activation of a buffer resulted in a significantly higher percentage viable bacteria compared to the other groups. It also resulted in a significantly lower percentage of nonviable bacteria compared to the NaOCl group and the control. Bacteria with compromised cell walls are probably more prone to be removed by the mechanical effect of a strong focused irrigant flow as their bond with the surrounding biofilm structure is weaker (Petridis *et al*. 2019b). Furthermore, the ultrasound group resulted in a significantly lower percentage of polysaccharidic content than NaOCl and RISA. Probably matrix polysaccharides, in contrast to viable bacteria, are easier to remove by focused fluid flow. In the root canal model, ultrasound was also more effective than the other groups in removing polysaccharides from the artificial lateral canal structures. Considering dentinal tubules as significantly smaller artificial lateral structures, this finding is not surprising.

In the present study, *E. faecalis* bacterial survival was possible, even without providing extra nutrition to the post‐treatment remaining bacteria. Intriguingly, the bacterial and polysaccharidic composition of the remaining biofilms after 5 days was not different at all in the RISA group and almost no different in the NaOCl group. This was in contrast to the groups in which buffer was applied either with syringe or with ultrasound. When buffer was applied with syringe, the remaining biofilms after 5 days exhibited increased polysaccharidic content compared to the post‐treatment remaining biofilms. When applied with ultrasound, less viable cells were noted compared to the post‐treatment remaining biofilm. This implies that ultrasonic activation of the buffer may have a prolonged effect over time. Chemical agents can alter the mechanical properties of the EPS, which is attributed to the effect these agents exert on the EPS network formation (Korstgens *et al*. 2001). This alteration can hamper biofilm removal if the matrix is stabilized, as for instance after chlorhexidine application (Brindle *et al*. 2011, Busanello *et al*. 2019). NaOCl also causes alterations in the viscoelastic properties of remaining biofilms which could render their subsequent removal even more difficult (Busanello *et al*. 2019). The effect of RISA is partly based on an osmotic action which could have resulted in a more or less ‘stabilized’ biofilm.

In the dentinal tubule model, the percentage of polysaccharides in the matrix was also analysed, in contrast from other studies evaluating intratubular decontamination (Arias‐Moliz *et al*. 2014, Arias *et al*. 2016). A significant increase in the matrix production was seen in the post‐treatment remaining biofilm after irrigation with NaOCl and after buffer irrigation, which is for both probably related to the stress of the environmental conditions. For the former an indication for the highest chemical stress (lowest amount of viable cells) and for the latter an indication for the most viable remaining biofilm reacting to environmental stress (no nutrition). Biofilm matrix plays an important role in the protection of biofilm bacteria against chemical and mechanical stresses (Flemming & Wingender 2010) and consequently a crucial role in *E. faecalis* survival (Lei *et al*. 2016). As a matter of fact, quantification of matrix material is a relevant approach in the search for what really happens with biofilm structure when it is in contact with the irrigating solutions in dentinal tubules (Lei *et al*. 2016). Moreover, during the initial bacterial attachment and colonization on a substrate by the planktonic cells (Cerca *et al*. 2005), the matrix can digest enzymes surrounding bacteria, making the contact between the microorganisms and the antibacterial agents more difficult (Bowen & Koo 2011). In the presence of matrix material, *E. faecalis* can, even in the nonculturable state, survive in the dentinal tubules (Trevors 2011, Lei *et al*. 2016), emphasizing the importance of matrix material.

### Trends emerging from the findings of the different experimental models

Some interesting correlating trends were identified. In the models where the contact surface irrigant–biofilm was relatively big, as for instance in the artificial isthmus structures and dentine discs, buffer appeared less effective than RISA and RISA less effective than NaOCl in removing biofilms. This trend was also observed in the dentinal tubule model, where significantly less viable bacteria were seen in the NaOCl group compared to RISA and buffer. However, this trend was reversed after irrigation stage 2 in the artificial isthmus structures. In stage 2, the capacity of the irrigants to remove remaining biofilms from stage 1 is evaluated. Here, more biofilm was removed in the ultrasound and buffer groups than in the RISA group, whilst RISA removed more biofilm than NaOCl. An explanation for that could be that the initial biofilm exposure to RISA and NaOCl induce structural alterations that render further removal of the remaining biofilms difficult. This has already been described for NaOCl (Busanello *et al*. 2019). This coincides with the findings of this study about the composition of the remaining biofilms that survived after 5 days, which was identical for RISA and NaOCl groups, but different for the buffer and ultrasound group. Moreover, further biofilm removal can be impeded by the chemical‐induced biofilm dormancy and the presence of bacterial ‘persisters’ in the biofilm (Koo *et al*. 2017). Furthermore, biofilms can survive NaOCl (Stewart *et al*. 2001) resulting in post‐treatment biofilm persistence (Nair *et al*. 2005, Ricucci & Siqueira 2010). Depending on the environmental conditions, the remaining biofilm can regrow (Chávez de Paz *et al*. 2008, Shen *et al*. 2010, 2016, Ohsumi *et al*. 2015) sustaining periapical disease (Siqueira & Rôças 2008). Therefore, investigating aspects of its structure could aid in the development of effective removal regimes (Peterson *et al*. 2015). However, in the dentine disc model, buffer, NaOCl and RISA did not influence the viscoelastic properties of remaining biofilm. Sodium hypochlorite exhibits limited penetration due to the immediate NaOCl consumption related to its reaction with the organic biofilm substrate (Stewart *et al*. 2001, Stewart 2003), an effect more pronounced when low NaOCl concentrations are used. In the present study, the biofilms grown on the dentine discs had a bacterial dense structure, which is low in water and EPS content (Busanello *et al*. 2019). The dense bacterial aggregation and the low water content may interfere with the mass transfer of solutes, which in turn also limit NaOCl penetration. This could also be the case for RISA and buffer irrigants. This biofilm could also be denser in structure than the biofilm of *E. faecalis* used in the dentine tubule model.

### Clinical translation of the laboratory experiments

For the root canal and dentine disc models, a bacterial dense biofilm has been used to more closely resemble the ground (basal) layer of the biofilm and a biofilm packed in narrow structures like lateral morphological features of the root canal. It can be concluded that for this type of biofilm more time than 30 s exposure for an effective chemical effect of NaOCl and RISA to develop. However, the alterations on the post‐treatment remaining biofilm matrix induced by the contact with the chemical agents could hamper further biofilm removal (Korstgens *et al*. 2001, Brindle *et al*. 2011, Busanello *et al*. 2019). Furthermore, the complex root canal anatomy further impedes biofilm removal. Remaining biofilms after treatment can eventually survive. Therefore, a strong chemical attack at the end of the root canal treatment procedures could be advised in order to effectively disturb any residual biofilm. Finally, these findings suggest that a final syringe irrigation with high flow rate could prove very effective in removing previously chemically affected biofilms.

## Conclusions

Within the limitations of the present study, the mechanical effect of syringe irrigation at low and high flow rate was important for biofilm removal. The mechanical effect of the lateral flow generated by ultrasound was effective when the surface contact biofilm–irrigant was small (artificial lateral canals). The mechanical effect of ultrasound in removing biofilm from an artificial isthmus structure was not more effective than syringe irrigation with a low flow rate.

After irrigation with NaOCl, significantly a lower amounts of viable bacteria were present in the dentinal tubules. After the different irrigation procedures, bacteria and/or biofilm remained in the dentinal tubules showing its ability to survive after a period of 5 days in the root canals without extra nutrition. Both RISA and NaOCl seemed to induce alteration on the remaining biofilm that hamper further biofilm removal.

## Conflict of interest

LWMvan der Sluis is coinventor of the intellectual property which is described in the pending patent: Composition, used, for example, as disinfectant or antimicrobial in dental treatment (e.g. root canal treatments), comprises at least one tonicity agent (organic acids and/or their salts e.g. sodium acetate) rendering the composition hypertonic. The patent is owned by University of Amsterdam, Amsterdam, the Netherlands. Patent Numbers: WO2011102724‐A2; NL2004260‐C; WO2‐011102724‐A3; US3012328708‐A1; EP2536378‐A2; CN102985051‐A. The authors have stated explicitly that there are no conflicts of interest in connection with this article.
